# Advances in Proteasome Enhancement by Small Molecules

**DOI:** 10.3390/biom11121789

**Published:** 2021-11-30

**Authors:** Dare E. George, Jetze J. Tepe

**Affiliations:** Department of Chemistry and Pharmacology & Toxicology, Michigan State University, East Lansing, MI 48824, USA; georg186@msu.edu

**Keywords:** proteasome, neurodegeneration, cancer, ubiquitin, 20S, 26S, misfolded, disordered, degradation, protein

## Abstract

The proteasome system is a large and complex molecular machinery responsible for the degradation of misfolded, damaged, and redundant cellular proteins. When proteasome function is impaired, unwanted proteins accumulate, which can lead to several diseases including age-related and neurodegenerative diseases. Enhancing proteasome-mediated substrate degradation with small molecules may therefore be a valuable strategy for the treatment of various neurodegenerative diseases such as Parkinson’s, Alzheimer’s, and Huntington’s diseases. In this review, we discuss the structure of proteasome and how proteasome’s proteolytic activity is associated with aging and various neurodegenerative diseases. We also summarize various classes of compounds that are capable of enhancing, directly or indirectly, proteasome-mediated protein degradation.

## 1. Introduction

The degradation of proteins is a continual process that is highly regulated by the two major proteolysis systems, the lysosomal degradation pathway and the proteasome-mediated pathway. Protein degradation helps maintain biological homeostasis in cells which are needed for all cell functions and for maintaining optimal conditions for enzyme function [1]. The proteasome pathway is the major pathway for the degradation of misfolded, oxidatively damaged, and redundant proteins. Dysregulation of proteasome function has been identified in the pathogenesis of several neurodegenerative diseases including Parkinson’s disease (PD) [2], Alzheimer’s disease (AD), and other neurodegenerative diseases [3]. The proteasome pathway is also involved in the regulation of several other cellular processes such as cell cycle, stress signaling, gene expression regulation, inflammatory response, cell differentiation, and apoptosis, which makes it an appealing target in the treatment of other types of diseases, including cancer [4]. Due to the critical role of the proteasome-mediated degradation pathway in cell regulation, the modulation of proteasome proteolytic activity has become a valuable strategy in the pursuit of new therapeutics to treat several neurodegenerative diseases [5,6,7,8].

### 1.1. The Human Proteasome

The human proteasome is a large complex protein responsible for the intracellular degradation of unwanted and damaged proteins via a ubiquitin-dependent and ubiquitin-independent degradation pathway. The most common proteolytic clearance of proteins proceeds by tagging the protein with polyubiquitin, after which it is degraded into small peptides of seven to eight amino acids by the 26S proteasome [9]. Highly disordered proteins can also be degraded in a ubiquitin-independent manner by the 20S proteasome [10]. In this review, we will cover the use of small molecules to enhance the proteolytic activity of both the 26S proteasome and the 20S proteasome.

### 1.2. Ubiquitin-Proteasome System

#### 1.2.1. Ubiquitin

**Figure 1:**
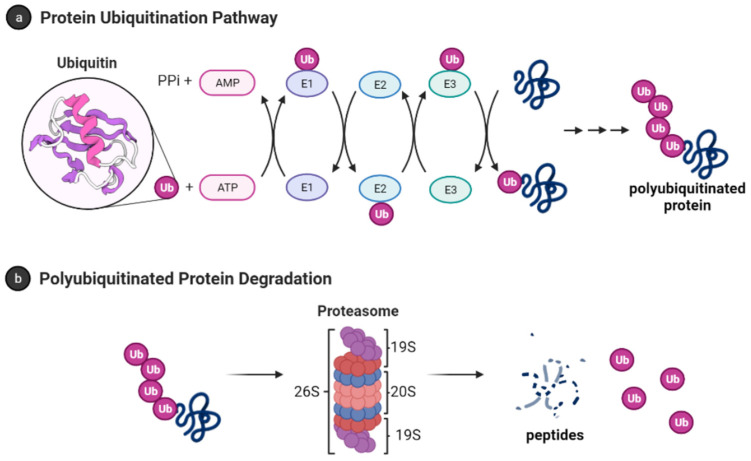
Ubiquitin-proteasome system [13]. (**a**) Protein polyubiquitination process using the ubiquitin-activating enzyme E1, conjugating enzymes E2 and the E3 ligase; (**b**) Polyubiquitinated proteins are degraded by 26S proteasome into small peptides following its deubiquitination.

Ubiquitin (Ub) is a small protein (approximately 8600 Da) with 76 amino acid residues responsible for tagging a wide range of cellular proteins for proteolytic degradation. In the ubiquitin-proteasome system (UPS) (Figure 1), proteins are tagged for proteolysis by covalent ligation to ubiquitin [11]. Ubiquitination of proteins requires three enzymes in chronological order (see Figure 1a). The E1 ubiquitin-activating enzyme, just like its name, activates the C-terminal glycine residue of the ubiquitin in an ATP-dependent manner. The binding of the ubiquitin to a cysteine residue of E1 forms a Ub-E1 complex via a thioester linkage. The E2 ubiquitin-conjugating enzymes transfer the ubiquitin from the Ub-E1 complex to itself via a trans-thioesterification to form the Ub-E2 complex and release the E1 enzyme from the system. Lastly, the ubiquitin ligases E3s are responsible for selecting proteins for ubiquitin-mediated proteolysis. Humans have two E1 enzymes, about 40 E2 enzymes, and are estimated to have about 500–1000 E3s [12].

After monoubiquitination of the targeted protein, the C-terminus of each ubiquitin molecule can be linked to any of the other seven lysine residues (K6, K11, K27, K29, K33, K48, and K63) on the previous ubiquitin to extend the ubiquitin chain and form the polyubiquitinated tagged protein [14,15]. However, the signal for protein degradation by the proteasome usually involves the linking of Ub to the K48 of the previous Ub on the protein [16,17]. In addition, K11, K29, and K63 linked chains have also been shown to play a role in proteasomal degradation [17,18]. The 26S proteasome degrades polyubiquitinated proteins (see Figure 1b), and a previous study shows that proteins marked for degradation must be tagged with at least four ubiquitin molecules to be recognized by the 26S proteasome [16,19]. However, shorter chains, monoubiquitinated and multiple monoubiquitinated proteins can also be targeted for degradation by the proteasome [20,21,22,23]. It is also important to note that the ubiquitination process is reversible, and the deubiquitinating enzymes (see Section 3.1.1) are present in the cell to remove ubiquitin-tagged proteins [24].

#### 1.2.2. The 26S Proteasome

The 26S proteasome has a molecular weight of approximately 2.5 MDa and it is made up of the 20S core particle (CP), and one or two 19S regulatory particle(s) (RP) attached to one or both end(s) of the CP [25]. The 19S RP (also known as PA700) binds to the 20S CP and facilitates the gate opening of the CP for proteolytic degradation of polyubiquitinated proteins [26]. The 19S RP is also responsible for recognizing, unfolding, and translocating polyubiquitinated protein into the 20S CP [27]. Cryo-EM studies have shown many conformation states of the 26S proteasome when engaged with substrate [28,29,30,31,32,33,34,35]. Some of these studies showed the processes by which substrate is engaged, deubiquitylated, unfolded, and translocated by the proteasome [28,29]. The proteasome is also referred to as the 30S proteasome when the 20S CP is capped at both ends with the 19S RP [36]. However, in this review, we will refer to the 26S proteasome without distinguishing between the singly or doubly capped CP.

### 1.3. The 20S Proteasome or Core Particle

The 20S proteasome is a 700 kDa protein with a cylindrical-like structure. The CP contains four heptameric rings stacked on each other in an α_1-7_β_1-7_β_1-7_α_1-7_ fashion. The outer α-rings form a gate, and they recognize regulatory particles that allow the opening and closing of the gate [37]. The inner β-rings contains six proteolytic sites, three on each β-ring (β1, β2, and β5), and are responsible for the proteolytic activity of the proteasome.

The three different proteolytic sites of the 20S CP exhibit different substrate preferences even though they all use N-terminal nucleophilic threonine to carry out their proteolytic activities. The β1 exhibits a caspase-like (C-L)/PGPH (peptidylglutamyl-peptide hydrolyzing) activity and preferentially cleaves after acidic residues. The β2 and β5 exhibit trypsin-like (T-L) and chymotrypsin-like (CT-L) activities, and they preferentially cleave after basic and hydrophobic residues, respectively [38]. The 20S proteasome on its own degrades unstructured proteins using a ubiquitin-independent pathway.

### 1.4. Small Molecule Regulation of Proteasome Function

Due to the role of the proteasome in cellular functions, the regulation of proteasome has become a valuable target for the development of therapeutic molecules [39]. Proteasome inhibition is a therapeutic approach for the treatment of cancer. For example, bortezomib, a dipeptide boronate, was approved by the FDA in 2003 as an anticancer drug to treat mantle cell lymphoma and multiple myeloma [40,41]. Bortezomib inhibits the 26S proteasome by forming a covalent bond between its boron atom and threonine oxygen in the CT-L catalytic site of the 20S CP [40]. Molecules that inhibit the proteasome have also been shown to induce apoptosis in cell cultures and murine models of cancer. One of the proposed mechanisms is that proteasome inhibition prevents the degradation of the I*κ*B, an NF-*κ*B inhibitor, which prevents NF-*κ*B nuclear translocation and consequently NF-*κ*B mediated gene expression [42]. Proteasome inhibition results in the accumulation of I*κ*B [43,44,45,46,47,48], cyclin-dependent kinase (CDK) inhibitor p21 [43,49,50], tumor suppressor p53, and other pro-apoptotic proteins [51,52,53]. The exceptional increase in apoptosis of certain multiple myeloma cells when treated with proteasome inhibitors has also been linked to an increase in protein unfolding and increasing substrate load on the proteasomes [54,55]. In addition, proteasome inhibition leads to lethal shortage of amino acids in the cells, which are the building blocks for cells to make new proteins. This amino acid scarcity caused by proteasome inhibition results in increasing ER stress and cell apoptosis [56]. Many reviews on proteasome inhibition have recently been published [57,58,59,60,61,62,63,64,65,66], including a recent review by our group on natural products scaffolds as inhibitors of the proteasome [67].

Proteasome activation by small molecules is a proposed strategy for the treatment of age-related diseases and several neurodegenerative diseases such as Parkinson’s disease (PD), Alzheimer’s Disease (AD), Huntington’s Disease (HD), and Amyotrophic Lateral Sclerosis [8,68,69,70,71,72]. Increasing the proteolytic activity of proteasome enhances the degradation of specific intrinsically disordered proteins (IDPs) such as α-synuclein, β-amyloid, and tau, to mention a few, which are associated with the pathogenesis of these neurodegenerative diseases. This review will focus on the use of small molecule enhancers of proteasome-mediated proteolysis as a potential strategy for the treatment of various neurodegenerative diseases.

## 2. Proteasome Activity and Diseases

**Figure 2:**
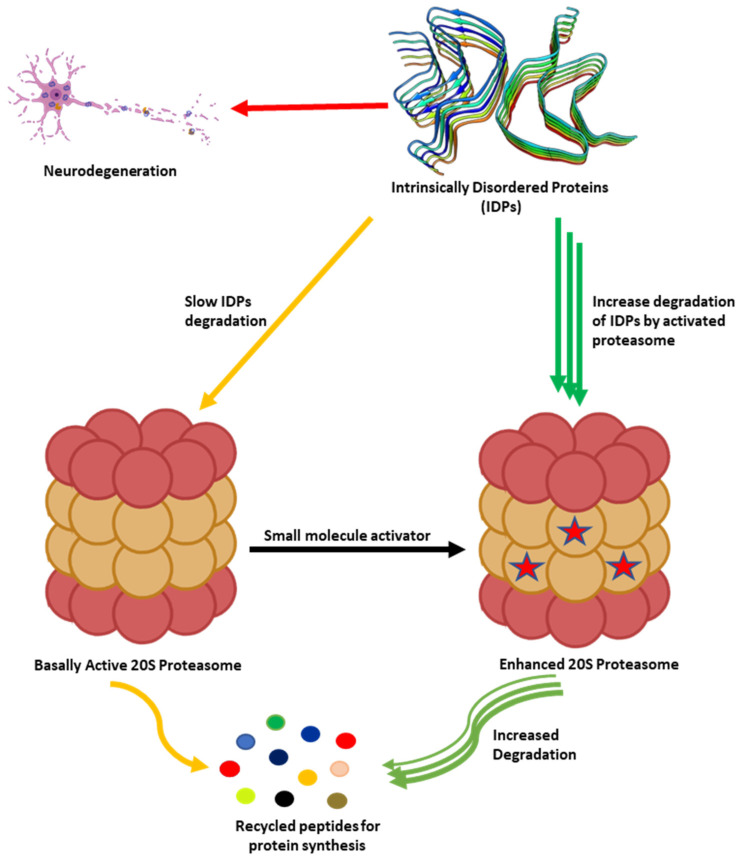
Accumulation of partially unfolded, misfolded, and dysregulated intrinsically disordered proteins (IDPs) such as amyloid-β and α-synuclein leads to neurotoxicity and neuronal cell death. The 20S proteasome degrades unwanted IDPs; however, small molecules can enhance the rate of proteasome-mediated degradation of these IDPs and prevent their accumulation.

As humans age, there is a decline in proteasome function [3,73]. This reduction could be due to the reduction in the expression of proteasome subunits [74], oxidative damage of the protein [75,76], and disassembly of the 26S proteasome holocomplex [77,78]. The decrease in proteasome proteolytic function leads to lower rates of unwanted protein degradation which can induce toxic signaling upon accumulation and aggregation (Figure 2). In particular, the accumulation of specific intrinsically disordered proteins (IDPs), such as amyloid-β and α-synuclein, have been identified as a driving cause of many neurodegenerative diseases [79,80,81,82,83,84,85,86,87,88,89,90,91,92,93,94,95,96,97,98]. The exact mechanism by which these oligomers induce neurotoxicity is complex and still debated, but it is widely accepted that dysregulated IDPs accumulate, and the resulting soluble oligomeric forms of these protein aggregates are likely toxic species in disease pathogenesis [79,93,99,100,101,102]. These soluble oligomeric forms are also responsible for impairing proteasome function, which further drives disease progression [94,103,104,105,106,107,108,109,110,111,112,113,114,115,116,117,118,119,120]. Multiple studies have indicated that enhancing proteasome proteolytic activity prevents the accumulation of these IDPs, reduces brain damage and improves cognitive performance in mouse models, and may be a new therapeutic strategy to treat neurodegenerative diseases [8,68,69,70,72,119,121,122,123,124,125,126,127,128,129,130,131,132]. More recently, it has been recognized that the 20S proteasome of the proteasome plays a critical role in maintaining proteostasis by the direct degradation of oxidatively damaged and highly disordered proteins [10,133,134,135,136,137,138]. The 20S proteasome, therefore, serves as the default protease to unremittently maintain low levels of these unwanted IDPs without the need for post-translation modifications, including protein ubiquitination [10,133]. Highly disordered proteins appear to be the main target of the 20S proteasome [139]. IDPs are also naturally short-lived, but basal levels are secured by forming proteolytically stable structured complexes with “nannies”, chaperones, or other protein complexes [140]. However, when these IDPs production outpaces their degradation, they accumulate, oligomerize, and aggregate, resulting in the induction of downstream cytotoxic signaling events. 

### 2.1. Aging

During aging, proteins are more susceptible to several types of modification, such as oxidation, glycoxidation, glycation, conjugation with peroxidation products, etc. These protein modifications can lead to decreased enzyme activity and thermodynamic stability [141,142], resulting in the accumulation of damaged proteins in the cell. Several models used to study proteasome proteolytic activity showed a decline in proteasomal activity as we age [143,144] and decreased degradation of oxidized proteins in cell cultures [145,146]. Reactive oxygen species accumulate during aging, resulting in an increase of oxidatively damaged proteins and an increased demand on the proteasome degradation system to eliminate these pathogenic aggregation-prone proteins [113]. Unfortunately, proteasome proteolytic activity declines as we age [3,73,75], resulting in the accumulation of oxidatively damaged proteins [147]. 

These unwanted protein aggregates interact with the proteasome and further reduce its proteolytic capacity [104,105,106,107,111,113]. This inhibition of proteasome leads to a compounding accumulation of more unwanted proteins and a vicious cycle of progressively worsening aggregation of oxidatively damaged proteins [94,104,105,106,107,116,117,118,119,148,149,150].

### 2.2. Neurodegenerative Diseases

#### 2.2.1. Parkinson’s Disease (PD)

Approximately 10 million people worldwide are affected by PD, making it the second most prevalent neurodegenerative disorder [151]. PD is characterized pathologically by the loss of dopaminergic neurons as a result of the accumulation of Lewy bodies in the substantia nigra pars compacta (SNc) [152]. Lewy bodies are the defining pathological hallmark of PD, and its major components are α-synuclein, ubiquitin, parkin, proteasomal components, and other UPS-related proteins. PD has been linked to various UPS proteins such as parkin and UCH-L1. Additionally, the expression of mutant α-synuclein in rat cells inhibits proteasome proteolytic activity, causing essential features common to PD such as inclusion body formation, accumulation of undegraded ubiquitinated protein, and cell death. Dysregulation of proteasome-mediated protein degradation has been associated with both familial and sporadic PD [153]. 

Different approaches have been used to determine the role of the proteasome in the pathology of PD. Rat models have been developed to display characteristics such as bradykinesia, tremor, and abnormal posture, which are similar to PD when treated with proteasome regulators [154]. In addition, α-synuclein and ubiquitin-containing inclusion resembling Lewy bodies were also present at the neurodegenerative sites of the rat neurons. However, other studies could not reproduce similar output [155], which became controversial and indicated that proteasome inhibition is not a reliable model to study PD. As an alternative approach to study the development of PD, mouse models of proteasome subunits knock-out were generated. However, the removal of most proteasome genes causes embryonic lethality except for a few immune-related subunits [109,156]. The deletion of the proteasomal ATPase subunit Psmc1/Rpt2 in the dopaminergic neurons leads to intraneuronal α-synuclein and ubiquitin-positive inclusion, which resulted in neurodegeneration and thus resembling the human PD. This study provided direct support for the involvement of neuronal proteasome and Lewy-like inclusion seen in PD [157].

#### 2.2.2. Alzheimer’s Disease (AD)

AD is the most common cause of dementia, and it is ranked as the sixth leading cause of death in the United State as of 2019 [158]. AD is associated with loss of cognitive functioning such as memory, thinking, and reasoning. It also impacts behavioral activities such as the ability to carry out daily life activities [159]. 

The pathogenesis of AD has been attributed to protein misfolding and aggregation [80,81,82,84,89]. It is characterized by the aggregation of extracellular β-amyloid plaques and intracellular accumulation of neurofibrillary tangles [160]. The neurofibrillary tangles (NFTs) are mostly composed of hyperphosphorylated microtubule-associated tau. Filamentous tau formation is triggered due to changes in the concentration of β-amyloid [160]. Although β-amyloid appears to be more specific to AD, tau is also associated with other neurodegenerative diseases such as corticobasal degeneration, chronic traumatic encephalopathy, argyrophilic grain disease, and progressive supranuclear palsy [161]. 

Different experimental and clinical data have shown that the main drivers of synaptic dysfunction, cognitive decline, and neuronal loss in AD patients are associated with soluble toxic β-amyloid oligomers which impair proteasome proteolytic activity, rather than insoluble β-amyloid plaques [111,162,163,164,165]. 

The ubiquitin-dependent proteasome system is associated with AD and degradation of β-amyloid [120,166,167,168]. Studies showed that the activity of the proteasome decreases in some parts of the brain in AD patients [169]. Similarly, inhibition of the 26S proteasome by lactacystin resulted in the accumulation of β-amyloid in both astrocytes and neurons, suggesting that β-amyloid could be a substrate for proteasomal degradation [166]. These results indicate that enhancing proteasome proteolytic activity may alleviate some of the factors that drive the pathogenesis of AD.

#### 2.2.3. Huntington’s Disease (HD)

HD is a brain disorder caused by the mutations of the huntingtin (Htt) gene. HD affects mood, movement and also leads to progressive cognitive deterioration and psychosis as a result of changes in the central part of the brain. The disease is dominant, which implies that it is inheritable by children from their parents.

In HD the disorder of polyglutamine in the Htt protein results in toxic functions of mutant Htt, which consequently leads to neurodegeneration. The HD mutation is an unstable expansion of trinucleotide CAG repeats within the Htt gene, which causes polyglutamine stretch in the N-terminal of the protein and results in the formation of fibril and aggregates [170]. Remarkably, the mutant Htt still retains some of the functions of a normal Htt. The number of CAG repeats correlates to the progression of HD and the symptoms. Individuals with 36–40 CAG repeats may or may not develop HD symptoms. However, those with CAG repeats above 40 will eventually develop HD [171,172]. Greater than 50 long CAG repeats cause early onset of the disease (juvenile HD) [173]. Several studies suggest that mutant Htt aggregates impair the ubiquitin-proteasome system [104,116,174]. However, the actual mechanism of interaction between the mutant Htt aggregate and the proteasome remains unclear. Interestingly, unlike the soluble Htt, the Htt aggregates have been found to be ubiquitinated, and those insoluble aggregates have also been shown not to impair the activity of the 26S proteasome [175,176]. The inclusions associated with HD have been proposed to be toxic and lead to neuronal death. But the exact mechanism of toxicity remains unsolved. Increasing proteasome-mediated substrate degradation has been shown to increase survival in HD patients’ mutant huntingtin-expressing striatal and skin fibroblasts neurons. Over expression of PA28γ, a proteasome activator subunit also improved cell viability [177].

#### 2.2.4. Amyotrophic Lateral Sclerosis (ALS)

ALS is another progressive neurodegenerative disease that affects the motoneurons in the brain and spinal cord. ALS is characterized by spasticity, muscle weakness, atrophy, and paralysis. The disease is often lethal within three to five years after diagnosis [178,179,180]. Like other neurodegenerative diseases, most ALS cases are sporadic (sALS), while about 10% could be familial (fALS) and result as a mutation in multiple genes [178]. Also, both sALS and fALS are clinically indistinguishable. Efforts at determining the mechanism underlying different fALS forms are thought to give insight into target identification and therapeutics development for both forms of diseases. 

The first ALS-linked genetic mutation was found in 1993, and it was located in the gene coding of Cu-Zn superoxide dismutase 1 (SOD1) [181]. Since then, several ALS mutant genes have been identified [178,182,183,184]. The recently discovered C9orf72 gene mutation has been identified as the most common cause of fALS and frontotemporal dementia [185,186]. An expansion of a GGGGCC repeat in the C9orf72 gene translates into five dipeptide-repeats proteins: poly-GA, poly-GP, poly-GR, poly-PR, and poly-PA [187,188,189,190,191]. A study showed that poly-GA aggregates recruit numerous 26S proteasome complexes which may affect neuronal proteasome-mediated proteostasis and the protein degradation process [192].

Both the sALS and fALS are often considered proteinopathies since they both tend to aggregate and accumulate misfolded and abnormal proteins generated in the damaged neurons [193,194]. The presence of ubiquitinated rich protein inclusions in motor neurons is a feature considered a common hallmark of not only human ALS but also in cellular and animal models of the disease [195,196]. The abundant accumulation of these ubiquitinated proteins suggests a significant contribution of the ubiquitin-proteasome system in these neuropathological features. In addition, the use of different cellular and animal models of ALS has provided substantial evidence of the involvement of the ubiquitin-proteasome system in the formation of inclusion and neuronal death [197].

## 3. Small Molecule Enhancers of 26S Proteasome Activity

The role of the proteasome in the regulation of cellular functions has made it an important target for the development of new treatments for cancer and neurodegenerative diseases. In addition, understanding proteasome regulation has allowed scientists to probe the mechanism of different cellular processes that involve the proteasome.

Small molecules that directly activate the 26S proteasome are rare and most of the well-studied approaches to enhance the 26S proteasome involve indirect activation by modulation of post-translational modification and by genetic manipulation. A recent review highlights some of the cellular mechanisms that activate 26S proteasomes [198]. In this review, we will focus on small molecules that activate the 26S proteasome.

### 3.1. Indirect Activation of 26S Proteasome

#### 3.1.1. Inhibition of Deubiquitinase

Deubiquitinating enzymes (DUBs) play a critical role in the ubiquitin-proteasome system (UPS). The 19S RP of the proteasome uses deubiquitinase activity to remove and recycle polyubiquitin from protein substrates that are condemned for proteolysis [199]. There are three essential DUBs: RPN11, UCH37, and USP14 that are associated with the 19S RP of human proteasome [124,200,201]. The main function of these DUBs is to remove monoubiquitin and polyubiquitin chains from substrates tagged for proteasomal degradation [202,203,204,205]. USP14, a DUB of the cysteine protease class, interacts reversibly with the proteasome [124,206,207,208] and it cleaves the ubiquitin chain off the targeted protein before degradation by the proteasome [209,210], thereby inhibiting the degradation of ubiquitin-protein conjugates in vitro and in vivo [124]. Unlike USP14, the RPN11, a DUB of the metalloprotease class, is part of the 19S RP and cleaves the ubiquitin chain after degradation has been initiated by the proteasome [200]. The mechanism of action of UCH37 is not completely understood yet but this DUB could edit the ubiquitin chains and either prevent the protein from being degraded or enhance its degradation depending on the proteasome needs [200,211,212]. 

**Figure 3:**
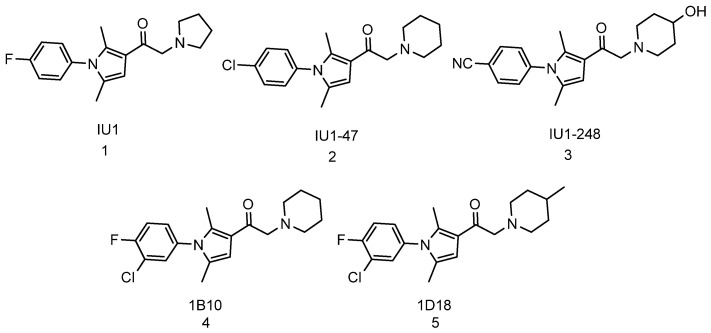
Small molecules inhibitors of deubiquitinase enzymes (DUBs).

A study conducted in 2010 showed that USP14 inhibits protein degradation by the proteasome in murine embryonic fibroblasts. In the same study, the authors showed that inhibition of USP14 by 1-[1-(4-fluorophenyl)-2,5-dimethylpyrrol-3-yl]-2-pyrrolidin-1-ylethanone (IU1, Figure 3, compound **1**) drastically stimulate the degradation of oxidized proteins by the proteasome [124]. IU1 was identified as a USP14 inhibitor from high-throughput screening (HTS) of over sixty-three thousand compounds for their ability to inhibit USP14. From the HTS, 215 compounds were identified as true inhibitors of USP14, however, screening of the hit compounds against several DUBs only provided three compounds as selective inhibitors of USP14. IU1 was found to be the most active of the three with IC_50_ of 4–5 µM [124]. Further optimization of IU1 has led to the discovery of more potent analogues such as IU1-47 (IC_50_ of 0.6 µM) (Figure 3, compound **2**) [213], IU1-248 (IC_50_ of 0.83 µM) (Figure 3, compound **3**) [214], and 1B10 and 1D18 (Figure 3, compound **4** & **5** respectively) which have better membrane permeability [215]. A recent review by Moon et al. [210], is focused on small molecules that inhibit proteasome-associated deubiquitinase and can be consulted for more information on DUB inhibitors. 

In a recent study by Kim et al. [216], proteasome-mediated proteolysis was increased by knocking down USP14 with small interfering RNA (siRNA) which led to a significant impairment of autophagic flux. This proteasome activation led to an increase in the microtubule-associated protein tau (MAPT) degradation and a decrease in the concentration of its oligomeric forms. This result is also consistent with Boselli et al.’s observation that USP14 inhibition enhances tau degradation in cultured neurons [213]. 

#### 3.1.2. Modulation of cAMP-Dependent Protein Kinase A (PKA) and cGMP-Dependent Protein Kinase G

Phosphorylation of proteasome subunits was recently established as a promising way to proteasome regulation [217]. The phosphorylation of Ser-14 of Rpn6, a subunit of 19S regulatory particle, by cAMP-dependent PKA has been shown to enhance the hydrolysis of polyubiquitinated proteins and small peptides in cells and in vivo studies [218,219,220,221]. In addition, impeding the phosphorylation of Thr-25 of Rpt3 by dual-specificity tyrosine-regulated kinase 2 (DYRK2) [222,223] and Ser-120 of Rpt6 by calcium/calmodulin-dependent protein kinase II (CaMKII) [224,225] or PKA [119] have been shown to impair proteasome proteolytic capacity and impedes cell proliferation. Small molecules that raise cAMP have therapeutical promise because they enhance the capacity of cell cultures [220] and mouse brains [119,226] to degrade misfolded proteins such as tau, which has been implicated in the pathogenesis of Alzheimer’s disease. 

**Figure 4:**
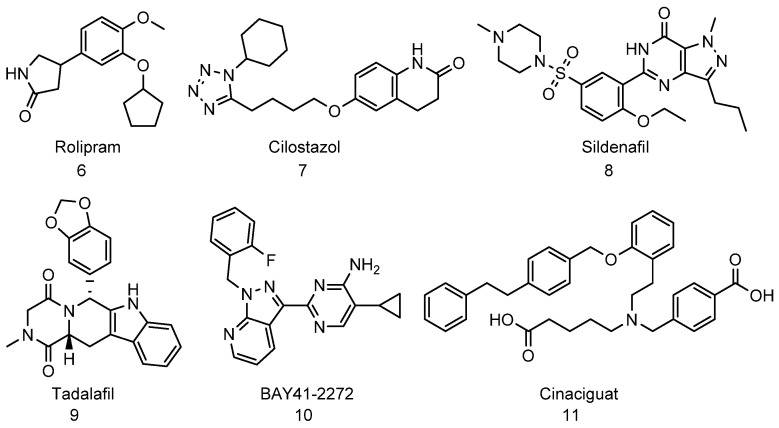
Rolipram (**6**) and Cilostazol (**7**) are examples of phosphodiesterase type-4 (PDE4) inhibitors. Molecules that inhibit PDE4 raise cAMP/PKA-mediated phosphorylation which increases the rate of degradation of IDPs and misfolded proteins in cellular assays and animal models. Sildenafil (**8**), Tadalafil (**9**), BAY41-2272 (**10**), and Cinaciguat (**11**) are molecules that raise cGMP level and induce cGMP-mediated proteasome activation.

Small molecule inhibitors of phosphodiesterase have been found to increase proteasome function by cAMP/PKA-mediated phosphorylation. Rolipram (Figure 4, compound **6**) is an example of phosphodiesterase type-4 inhibitor (PDE4) that was developed as an antidepressant drug in the early 1990s [227]. A study shows that Rolipram decreases the level of insoluble tau and improves cognitive performance in mice by increasing proteasome function through activating cAMP-PKA signaling [119]. Cilostazol (Figure 4, compound **7**) is another phosphodiesterase type-3 inhibitor (PDE3). Administration of Cilostazol in rTg4510 mice also showed improved cognitive performance and increased proteasome function through the cAMP/PKA pathway [226]. This small molecule was approved by the FDA to treat intermittent claudication and can also be used for secondary stroke prevention [228]. In late 2020, the FDA completed the clinical trial to determine the therapeutical use of cilostazol for patients with mild cognitive impairment [229].

Like cAMP-mediated modulation of 26S proteasome, small molecules that raise cGMP and activate PKG were recently shown to enhance proteasome proteolytic activity without affecting lysosomal degradation and increase the rate of degradation of both short-lived and long-lived proteins, including tau and mutant Htt. [198,230]. In the study conducted by VerPlank et al. [230], treatment of human neuroblast cells (SH-SY5Y) with molecules that raises cGMP such as sildenafil (Figure 4, compound **8**) or tadalafil (Figure 4, compound **9**) which are phosphodiesterase type-5 inhibitors (PDE5), or BAY41-2272 (Figure 4, compound **10**) and cinaciguat, (Figure 4, compound **11**) which are stimulators of soluble guanylyl cyclases, led to a rapid increase in proteasomal activity in cell lysates. However, unlike phosphorylation of Rpn6 by PKA [220,221], Rpt3 by DYRK2 [222,223], or Rpt6 by CaMKII) [224,225] or PKA [119], phosphorylation of Rpn6, Rpt3, or Rpt6 subunit was not observed in the PKG pathway [230]. Overexpression of PKG in SH-SY5Y and HEK293 cells led to an increase in the level of phosphorylated proteins compared to cells that were transfected with empty vectors during proteasome preparations. Thus, the 26S proteasome subunit or an associated protein that is phosphorylated in the cGMP-mediated proteasome activation is still unknown and the mechanism of action remains unclear.

#### 3.1.3. Inhibition of p38 Mitogen-Activated Protein Kinase (MAPK)

MAPKs are enzymes that phosphorylate the hydroxyl group of threonine and serine residues in proteins. These kinases play an important role in the control of cell proliferation and apoptosis. The p38 MAPK is involved in a signaling pathway that regulates various biological functions including biosynthesis of cytokinesis such as interleukin-1β (IL-1β) and tumor necrosis factor-α (TNF-α) [231,232]. The activation of the p38 MAPK pathway as a defense to osmotic stress has been shown to lead to phosphorylation of 19S RP at Thr-273 of the Rpn2 subunit, which resulted in the inhibition of the 26S proteasome proteolytic activity [233].

In ALS and AD, the over-activation of the p38 MAPK pathway has been reported in animal models and postmortem brains of AD patients [195,234,235,236]. The activation of the p38 MAPK pathway in cell lines and animal models has led to tau phosphorylation, neuroinflammation, neurotoxicity, and synaptic dysfunction, which are events associated with Alzheimer’s disease. Therefore, the search for p38 MAPK inhibitors became a novel approach for targeting neurodegenerative diseases [237].

**Figure 5:**
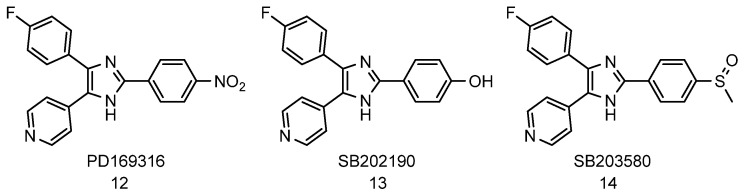
Imidazole inhibitors of p38 MAPK that enhances proteasome activities.

In 2017, Leestemaker et al. [122] discovered imidazole inhibitors of p38 MAPK as enhancers of 26S proteasome proteolytic activity. The compounds were identified from high-throughput screening of over 2750 compounds using a proteasome activity-based probe (Me_4_BodipyFLAhx_3_L_3_VS) that covalently binds to proteasome catalytic sites in an activity-dependent manner in living cells. The group found that PD169316 (Figure 5, compound **12**), a known inhibitor of p38 MAPK and its structural analogues, SB202190 (Figure 5, compound **13**), and SB203580 (Figure 5, compound **14**), increases the proteolytic activity of the proteasome in a dose-dependent manner in MelJuSo cells. Further characterization of these compounds showed that they increase proteasome proteolytic activity by inhibiting the p38 MAPK pathway without affecting cell viability, subunits abundance, and the overall level of ubiquitinated proteins [122]. Similarly, Huang et al. [238], demonstrated that treatment of HAP 40 depleted cells with p38 MAPK inhibitor, PD169316, increases the CT-L activity of the proteasome and enhances degradation of both soluble and aggregated forms of mutant Htt in a Huntington’s disease model. These data suggest that the regulation of the p38 MAPK pathway could be a potential way of modulating proteasome-mediated proteolytic activity.

#### 3.1.4. Proteasome Activation by Genetic Manipulation

Another approach to enhancing proteasome proteolytic activity is by proteasome subunit overexpression. Overexpression of β5i subunit in HeLa cells and lymphoblasts has led to an increase in the CT-L and T-L activities of the proteasome [239,240]. Previous studies also showed that stable overexpression of the β5 subunit in human fibroblast cell lines increased the level of other β subunits, increasing the overall proteolytic activity of the three catalytic sites [241]. Furthermore, overexpression of the 19S RP subunit PSMD11/Rpn6 increases proteasome assembly and proteolytic activity in human embryonic stem cells [242]. 

Small molecule activation of the transcription factor NRF2, nuclear factor erythroid 2-related factor 2, enhances the expression of the 20S and 19S proteasome particles and increases the proteolytic activity of the proteasome in cells containing NRF2. 18α-glycyrrhetinic acid (18α-GA) has been shown to increase proteasome proteolytic activities from 1.5- to 1.8-fold, with the activity of the caspase-like site being the most affected in wide-type HFL-1 human fibroblasts. In addition, the increase in the proteasome proteolytic activity was not observed when NRF2 was knockdown using siRNA in HFL-1 cells and the cells were treated with 18α-GA, further confirming the upregulation of proteasome proteolytic activity through NRF2 activation [243]. The activation of NRF2 increases the level of 20S proteasome subunits: α4, β1, β2, and β5 in both human fibroblasts [243] and mice liver [244], and the 19S subunits: Rpt2, Rpt5, and Rpn11 in mice liver [244]. The expression of antioxidant enzymes such as UDP-glucuronosyltransferase (UGT) [245], glutathione S-transferase (GST) [246], and NAD(P)H quinone oxidoreductase 1 [247], to name a few, are also controlled by this transcription factor. Activation of NRF2 by *tert*-Butylhydroquinone (t-BHQ) and sulforaphane increases proteasome proteolytic activity in human embryonic stem cells (hESCs) [248] and also protects against oxidative stress [249].

## 4. Small Molecule Enhancers of 20S Proteasome Activity

### 4.1. Sodium Dodecyl Sulfate (SDS)

SDS (sodium dodecyl sulfate), also known as SLS (sodium lauryl sulfate), is a synthetic organosulfate salt used in cleaning, pharmaceutical, and food products. In 1988, Tanaka et al. [250] showed that 20S proteasome proteolytic activity could be enhanced at a low concentration of SDS (0.04–0.08%) in biochemical assays. However, at higher SDS concentrations, the activity of the proteasome is lost, and the SDS inhibits the proteasome [251]. SDS is an invaluable in vitro tool that is used by most researchers to activate the proteasome as means to test compounds for subsequent proteasome inhibition. It is believed that SDS induces gate opening of the proteasome by partial denaturation of the 20S to facilitate substrate entrance into the catalytic core. However, the actual mechanism of SDS proteasome activation is still unclear and considering that SDS is a detergent, it should not really be considered as a small molecule activator of the 20S proteasome.

### 4.2. Natural Product-Based Activators

**Figure 6:**
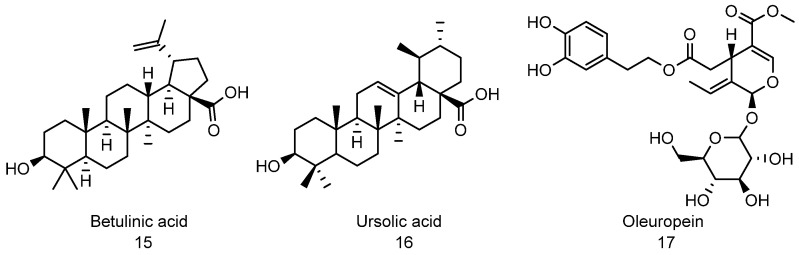
Natural product-based activators of 20S proteasome.

Several natural products have been identified as 20S proteasome activators, some of which include betulinic acid (Figure 6, compound **15**) [252], ursolic acid (Figure 6, compound **16**) [127], and oleuropein (Figure 6, compound **17**) [253]. Betulinic acid is a triterpene isolated from the bark of *Betula pubescens* (commonly known as white birch). It was reported as a selective inhibitor of human melanoma and it has been demonstrated to induce programmed cell death in human neuroblastoma and neuroectodermal tumor cells [254]. Betulinic acid is one of the first reported enhancers of the 20S proteasome. A small peptide assay using Suc-Leu-Leu-Val-Tyr-AMC (used to determine the CT-L activity of the proteasome) showed that’s betulinic acid enhances the CT-L activity of the proteasome with EC_50_ of approximately 2.5 μg/mL. Unfortunately, several chemical modifications to enhance the activity of betulinic acid resulted in compounds that inhibit the proteasome [252]. Like betulinic acid, ursolic acid is another triterpenoid that enhances the activity of the 20S proteasome. Ursolic acid is similar in structurally to betulinic acid, and they both enhance the CT-L activity of the 20S proteasome [127,252]. Although both betulinic acid and ursolic acid showed good activity in small peptide assay, unfortunately, betulinic acid did not show any activity for the turnover of misfolded proteins in vitro and in vivo [72].

In addition, other natural compounds have been identified as 20S proteasome agonists. Some of these compounds include lipids [255] and fatty acids [256]. In 1993, Ruiz de Mena et al. [255] studied the effect of phospholipids on the T-L, CT-L, and C-L activities of the proteasome in rat liver. In the study, the team identified cardiolipin (diphosphatidylglycerol) as a strong CT-L enhancer (up to 60-fold enhancement) and C-L enhancer (up to 30-fold enhancement). SDS and cardiolipin activation was shown to be additive and at either optimal or suboptimal concentrations of both compounds. Furthermore, fatty acids such as oleic, linoleic, and linolenic acids isolated from spinach leaves were found to increase proteasome-mediated substrate degradation by enhancing CT-L and C-L activities at about one-third to one-sixth the required concentration of SDS. Unlike SDS, at extremely low concentration (0.0007–0.0025%, ~25–90 µM), the T-L catalytic site is inhibited and the degradation of Boc-L-R-R-AMC is prevented [256].

### 4.3. AM-404 and MK-886

**Figure 7:**
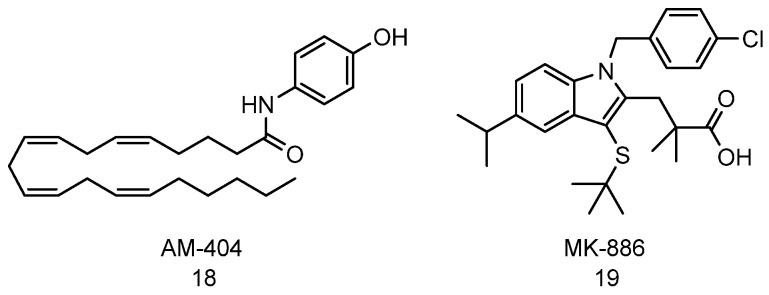
Structure of AM-404 and MK-886.

Although many compounds show increased peptide cleavage activities using the standard aminomethyl coumarin tagged small peptide substrates, most have failed to demonstrate an increase in proteolytic activity under physiological conditions. One likely explanation is that the small peptide probes, used for detection of in vitro proteasome proteolytic activity, may be small enough to inadvertently enter the CP-proteolytic cavity following minor conformational changes to the gate. Trader and Kodadek developed a follow-up assay that uses larger peptides with a single cleavage site and uses LC-MS to monitor proteasome proteolytic activity over time. They also validated molecules from LC-MS assay for their ability to turnover of α-synuclein in cells by monitoring the appearance of free GFP which correlates to the number of α-synuclein that was degraded. Using these assays, the lab was able to identify small molecules capable of increasing 20S mediated proteolytic activity [72]. The authors screened 726 compounds in the NIH Clinical Collection and identified AM-404 (Figure 7, compound **18**) and MK-886 (Figure 7, compound **19**) as “true” proteasome enhancers. The study showed both compounds increase the proteolytic activity of the 20S proteasome by 3- to 4-folds with an EC_50_ of 32 µM, and they also enhance the degradation of α-synuclein in cell culture [72]. 

Recently, the Trader’s lab investigated the structural component of AM-404 needed to enhance the proteasome proteolytic activity. In the study, they synthesized various derivatives of AM-404 by varying the aliphatic chain length, degree of unsaturation, and substitutions. They illustrated the importance of the aliphatic chain length and the *cis*-alkene at C8 of the aliphatic chain in stimulating the 20S proteasome [126].

### 4.4. Imidazolines

Imidazolines are an important class of compounds that are found in various natural and synthetic bioactive molecules [257,258]. This class of compounds displays a wide range of biological activities including proteasome and NF-*κ*B modulation [259,260,261], and therapeutic significance such as antifungi [262], antitumor [263], antihelminthics [264], antihyperglycemic [265], and antihypertensive activity [266].

**Figure 8:**
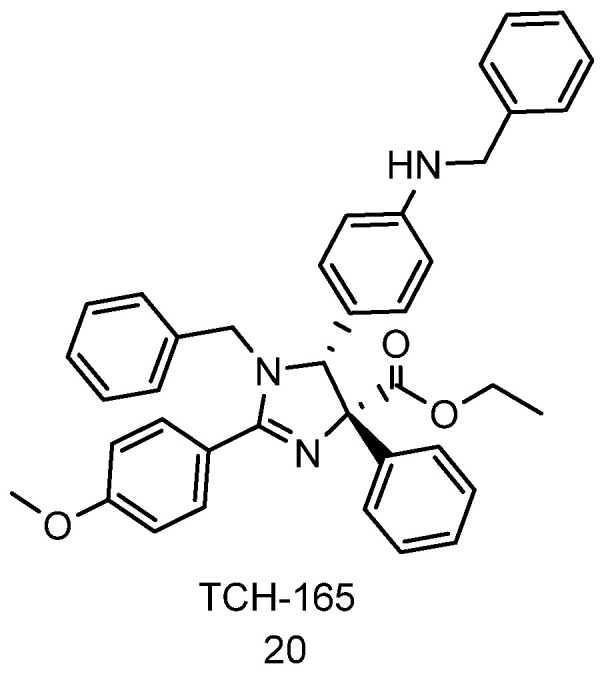
Imidazoline TCH-165 enhances proteasome activities and degrades intrinsically disordered proteins.

Our lab reported the imidazoline, TCH-165 (Figure 8, compound **20**), as a 20S proteasome enhancer as a low (1.5 μM) activator of the 20S proteasome [70]. TCH-165 enhanced 20S mediated degradation of IDPs such as α-synuclein, tau, ornithine decarboxylase, and c-Fos in cell cultures. However, it does not affect the degradation of structured proteins such as GAPDH. Treatment of HEK293T cells with TCH-165 showed a time-dependent disassembling of both the singly and doubly capped 26S proteasome and showed an increase in the free 20S CP. TCH-165 prevents the binding of the 19S RP to the 20S proteasome suggesting that the molecule binds directly on the α-ring of the 20S CP and shifts the equilibrium between 26S and 20S proteasomes towards an activated 20S CP. To gain insight into the mechanism of 20S proteasome activation, atomic force microscopy (AFM) imaging revealed that the ratio of open to closed 20S proteasome increases in a dose-dependent manner when treated with TCH-165 at concentrations as low as 200 nM [70]. This further supports that TCH-165 induces the open gate conformation of the 20S CP. It is also important to note that this is the only small molecule with biophysical data (atomic force microscopy (AFM) imaging) that support gate opening of the 20S proteasome [70].

### 4.5. Chlorpromazines

**Figure 9:**
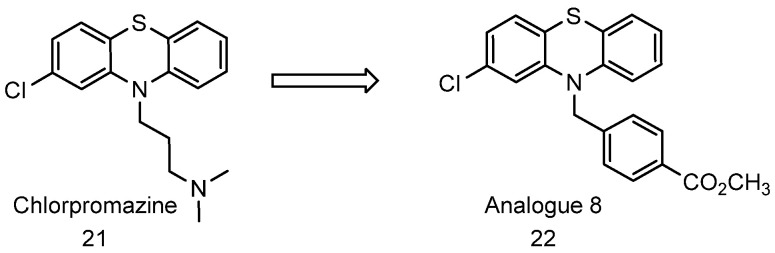
Structure of chlorpromazine and a chlorpromazine analogue **8**.

During the search for proteasome activators, our lab screened the NIH Clinical Collection and Prestwick libraries, where we identified chlorpromazine (Figure 9, compound **21**) and related phenothiazines as 20S proteasome activators inducing up to 20-fold activity [69]. Chlorpromazine is an FDA-approved drug that is used in the treatment of schizophrenia or manic-depression in adults. Chlorpromazine is believed to be a dopamine antagonist with some antiserotonergic and antihistaminergic properties [267]. 

Chlorpromazine and related phenothiazines preferentially enhance the CT-L activity of the proteasome and promote degradation of IDPs, such as *α*-synuclein and tau but not structured proteins in in vitro assays. Chemical modification of chlorpromazine abrogated its dopamine D2R receptor activity while preserving its ability to enhance the 20S proteolytic activity. Analogue **8** (Figure 9, compound **22**), an analogue of chlorpromazine with physiological insignificant potency for dopamine receptor (*K_i_* ≥ 250 µM) showed better efficacy with about 10-fold maximum enhancement and EC_200_ (concentration where the 20S mediated proteolysis is increased by 2-fold or 200%) of 13.5 µM. 

Interestingly, a structural analogue of chlorpromazine, methylene blue, was also found to enhance the CT-L and T-L activity of the 20S proteasome. Methylene blue was also found to decrease the level of β-amyloid and increase learning and memory in 3xTg-AD mouse model but does not affect tau phosphorylation in vivo [268]. A recent study also showed that methylene blue inhibits caspase-6-induced neurodegeneration, decreases neuroinflammation, and prevents cognitive impairment in mice [269].

### 4.6. Dihydroquinazolines

The 3,4-dihroquinazoline compounds are found in several natural products and synthetic compounds with various biological properties. Members of this class of compounds have biological properties that includes antifungal [270], antiparasitic [271], antitumor [272,273,274,275,276,277,278], and antiviral activities [279,280].

**Figure 10:**
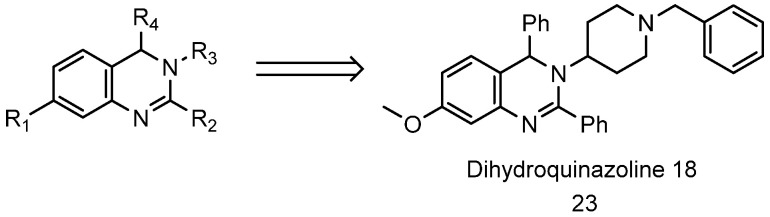
Structure of dihydroquinoline scaffold and dihydroquinoline **18** (compound **23**).

Earlier this year, Mosey et al. synthesized and evaluated several dihydroquinoline analogues as 20S proteasome enhancers [125,281]. In this study, they were able to identify several promising 20S activators with the most potent being dihydroquinazoline **18** (Figure 10, compound **23**), doubling proteasome proteolytic activity at 1.3 µM (EC_200_ 1.3 μM). The dihydroquinazolines enhance the three catalytic sites activity of the 20S proteasome and increase the degradation of α-synuclein, the IDP identified in the pathogenesis of Parkinson’s disease.

### 4.7. Fluspirilene and Acylfluspirilene

**Figure 11:**
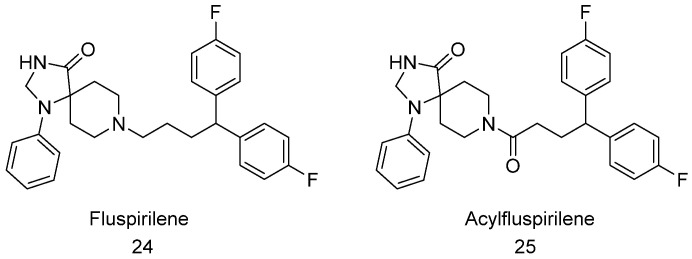
Structure of Fluspirilene and Acylfluspirilene.

Earlier this year, the Tepe group identified fluspirilene (Figure 11, compound **24**) and its synthetic analogues which were capable of enhancing 20S proteasome proteolytic activity and even restoring the proteolytic activity of 20S proteasome impaired by IDP oligomers [132]. Fluspirilene and its amide derivative, acylfluspirilene (Figure 11, compound **25**) activate the three catalytic sites of 20S CP and prevent IDP aggregation and oligomerization. Interestingly, acylfluspirilene exhibits more potency (EC_200_ 1.9 µM) compared to fluspirilene and a better maximum fold enhancement of greater than 20-fold. Furthermore, molecular docking shows that fluspirilene and acylfluspirilene bind to the α2-3 intersubunit pocket of the 20S CP, which is different from the previously reported 20S enhancers, TCH-165, dihydroquinoline, and chlorpromazine, which bind in the α1-2 pocket of the proteasome. In silico and in vitro structure-activity relationship (SAR) studies indicated the importance of the in-pocket binding interactions of these molecules with the 20S proteasome. This group of molecules does not enhance the proteolytic activity of the 26S proteasome and may therefore be used to selectively prevent the accumulation of dysregulated intrinsically disordered proteins without affecting regular ubiquitin-dependent protein degradation [132].

### 4.8. Pyrazolones

**Figure 12:**
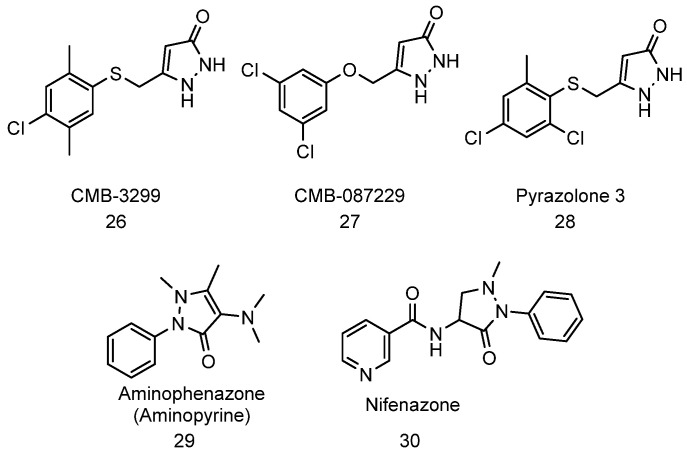
Structure of pyrazolones that have been shown to increase proteasome activities.

Pyrazolones are a rare class of compounds that enhance proteasome activation. This class of molecule was first discovered as proteasome activator in 2014 by the Silverman group and as potential compounds for the treatment of ALS [71]. The pyrazolones (Figure 12, compound **26**–**28**) were shown to protect neurons in PC12-SOD1^G93A^ cells in cellular models of ALS. The compounds also increased ALS transgenic mouse survival by 13%, further confirming their potential in the development of ALS therapeutics. During the mechanistic investigation of CMB-087229 (Figure 12, compound **27**), the compound was found to antagonize G protein-coupled receptor metabotropic glutamate receptor 5 (mGluR_5_), a previously identified target in ALS therapeutic [282], to about 65% at 10 µM concentration. The group investigated if mGluR_5_ was the target of the pyrazolones by screening known mGluR_5_ antagonists in their cell-based assay. However, the screened mGluR_5_ receptor antagonists (including MPEP and fenobam) showed no activity in the assay. Based on the result, it was concluded that the antagonism of the mGluR_5_ is unlikely to be the mode of action of the pyrazolones. Pull down experiments indicated several 26S proteasome regulatory subunits as a possible target for the pyrazolones. The pyrazolones were able to reverse bortezomib-induced cytotoxicity in the PC12 cells, further supporting evidence that their mechanism of action involved proteasome activation [283]. 

Following up on Silverman’s discovery, Santoro et al. [284] screened a small library of structurally-related pyrazolones for proteasome enhancement and neuroprotection against amyloid-induced toxicity in neuroblastoma SH-SY5Y cells. The group reported that the aminopyrine analogue (Figure 12, compound **29**) and nifenazone (Figure 12, compound **30**) displayed up to twofold induction of 26S proteasome proteolytic activity in cells. Using docking studies coupled with Saturation Transfer Difference (STD) NMR experiments, the group proposed that aminopyrine enhances the 20S proteasome by a mechanism involving binding to the α-ring surfaces of the proteasome; however, only a marginal increase in activity was observed (<30% increase at 10 μM) in a purified proteasome assay. 

## 5. Conclusions

Efficient proteasome function is critical in maintaining healthy cellular homeostasis. Dysregulation of protein or proteasome impairment can result in a toxic accumulation of unwanted proteins, which is observed in the pathogenesis of different neurodegenerative diseases and aging. Enhancing the proteolytic activity of the proteasome by increasing its capacity, accessibility, or the rate at which it degrades has long been hypothesized as a means to prevent the accumulation of dysregulated IDPs. More recently, researchers from various labs have explored the use of small molecules to induce protein proteolysis. Small molecule proteasome agonists can enhance the proteolytic clearance of unwanted proteins and restore homeostasis. Small molecule enhancers of the 26S proteasome are described herein which mainly induce enhanced 26S-mediated proteolysis of ubiquitinated proteins via an indirect mechanism of proteasome activation. 

Small molecule inhibitors of deubiquitinases prevent proteins marked for ubiquitin-dependent degradation fromescaping their fate. Even though there are no approved therapies yet based on deubiquitinating enzyme (DUB) inhibitors, this is an emerging field with great significance. Small molecule regulation of upstream signaling pathways, including cAMP-depending protein kinase A and c-GMP-dependent protein kinase G, affect the phosphorylation of the proteasome regulatory particles. As a result, small molecule regulators of phosphodiesterase type-3 (PDE3) can therefore indirectly increase the rate of substrate degradation by the proteasome. Small molecules that directly interact with the 26S proteasome and enhance the rate of 26S proteasome-mediated protein degradation are less known and likely a fruitful field for exploration. 

Whereas the 26S proteasome targets ubiquitinylated protein substrates, the 20S proteasome is limited to the degradation of only disordered proteins. Several small molecule enhancers of 20S proteasome-mediated protein degradation have been identified in the literature. We summarized herein several different classes of small molecule 20S proteasome enhancers that induce 20S—mediated degradation of dysregulated intrinsically disordered proteins by direct interaction with the 20S core particle. 

The activation of the proteasome by small molecules is a relatively new field in science. Its potential as a therapeutic approach is still unknown and the consequences of chronic exposure to proteasome enhancers are not known. However, considering the possibility of treating multiple disorders for which there are currently no treatment options available, this approach has enormous potential. However, as in all new fields, the approach still needs further validation, in vivo studies in particular, to fully understand its therapeutic potential and limitation. In addition, more studies are needed to elucidate the mechanistic details of small molecule proteasome activation and its overall cellular consequences.
